# Cheilitis: An unusual presentation of Mucocutaneous Leishmaniasis

**DOI:** 10.1002/ccr3.1609

**Published:** 2018-05-28

**Authors:** Vathulan Sujanitha, Thirunavukarasu Kumanan, Srisaravanapavananthan Felicia, Navaneethakrishnan Suganthan

**Affiliations:** ^1^ Teaching Hospital‐Jaffna Jaffna Sri Lanka

**Keywords:** cheilitis, endemic, leishmaniasis, mucocutaneous

## Abstract

The Mucocutaneous Leishmaniasis (MCL) involving lip is extremely uncommon. A clinical diagnosis of Leishmaniasis of the lips is often challenging to the treating clinician and may result in delayed diagnosis as this case illustrates. MCL should be considered in the differential diagnosis of lip lesions in a Leishmania endemic area.

## Graphical abstract

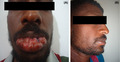

A 44‐year‐old previously healthy farmer from Mannar, an agricultural land of Northern Province of Sri Lanka presented with a history of progressively increasing swelling of lower lip for eight‐month duration. Examination revealed nontender, diffuse, smooth, and erythematous swelling of the lower lip with no lymphadenopathy. Investigations did not point toward any underlying systemic etiology.

What are your differential diagnoses at this point?

A clinical diagnosis of granulomatous cheilitis prompted a punch biopsy of the lower lip. The histology showed chronic inflammatory cell infiltrates with no granulomata. Patient was treated with a course of local and systemic steroids with no apparent clinical response.

The lesion was gradually getting worse over next few months with nodular lesions and ulcers (Figure 1A). A deep biopsy was performed at this juncture which showed dense infiltrate of chronic inflammatory cells including plasma cells and histiocytes with abundant amastigotes of Leishmania (3+) within the histiocytes. A PCR for Leishmania donovani was positive. Intramuacular sodium stibogluconate which is the sole available agent in the health sector was initiated according to WHO guidelines and continued for 21 days which led to complete resolution of the lesion (Figure 1B), and he was kept under close surveillance for a relapse. MCL of lip alone is an extremely rare presentation.^1^

**Figure 1:**
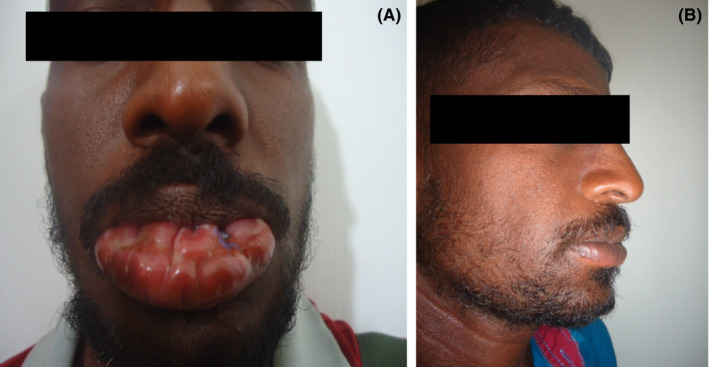
A, Swollen nodular ulcerated lower lip before treatment. B, Complete resolution after treatment

## CONSENT

Informed written consent was taken from the patient to use the images for the publication.

## CONFLICT OF INTEREST

The authors have no conflicts of interest to declare.

## AUTHORSHIP

SF: was the first contact of the patient and responsible for the management of the condition described. TK, VS, and NS: were the medical team involved in the evaluation to rule out the other medical conditions for the presentation. TK, VS, and NS: also involved in the preparation of the manuscript and finally approved by all authors.
